# A Tri-network Model of Human Semantic Processing

**DOI:** 10.3389/fpsyg.2017.01538

**Published:** 2017-09-12

**Authors:** Yangwen Xu, Yong He, Yanchao Bi

**Affiliations:** National Key Laboratory of Cognitive Neuroscience and Learning & IDG/McGovern Institute for Brain Research, Beijing Normal University Beijing, China

**Keywords:** dual coding, module, hub, embodiment, language, control

## Abstract

Humans process the meaning of the world via both verbal and nonverbal modalities. It has been established that widely distributed cortical regions are involved in semantic processing, yet the global wiring pattern of this brain system has not been considered in the current neurocognitive semantic models. We review evidence from the brain-network perspective, which shows that the semantic system is topologically segregated into three brain modules. Revisiting previous region-based evidence in light of these new network findings, we postulate that these three modules support multimodal experiential representation, language-supported representation, and semantic control. A tri-network neurocognitive model of semantic processing is proposed, which generates new hypotheses regarding the network basis of different types of semantic processes.

## Toward a network perspective of semantic processing

Semantic memory contains general knowledge about the world, including objects, people, facts, and beliefs, that is abstracted away from specific experiences (Yee et al., 2013) and is crucial to a wide range of human cognitive functions including language, memory, object recognition and use, and reasoning. Semantic knowledge can be obtained and stored in various ways. Consider the concept of “Beijing.” Someone who has never been in or seen anything about Beijing can deduce from linguistic contexts such as, “Beijing is the capital of China” that it is an important city that belongs to China. One may also know about Beijing by actually being there and experiencing it. These approaches of gaining knowledge about Beijing roughly correspond to two types of proposals about how semantic memory is developed and organized: one is based on experiences of various specific attributes, and the other is based on rich information supported by language, such as, word associations, word orders, and syntactic structures.

For the brain basis of semantic processing, decades of neuroimaging studies have consistently localized it to widely distributed brain regions across temporal, frontal, and parietal cortices (Binder et al., 2009). The conventional approach used by these studies is to identify regions activated by semantic tasks or lesion patterns associated with semantic deficits and to understand the function of each region in isolation. The prevailing models are dominated by the experience/attribute-based representation of semantics, interpreting the regions that loosely belong to the sensorimotor cortices as representing semantic attributes of corresponding modalities (e.g., form, color, motion, sound, action, and emotion; Martin, 2016). Language is often considered as a processing modality in parallel to these modalities rather than as a system that makes special contributions to semantic representation (Patterson et al., 2007; Lambon Ralph et al., 2016). Regions outside the modality-specific cortices or with functions related to semantic processing across modalities are often assumed to bind multiple attribute/modality-specific representations, e.g., the anterior temporal lobe (Patterson et al., 2007; Lambon Ralph et al., 2016) or the high-level convergence zones in the left temporal and inferior parietal regions (Binder and Desai, 2011), or to implement control processes that retrieve and manipulate semantic knowledge in a task- and context-appropriate fashion, e.g., the left frontoparietal and the left posterior temporal cortical areas (Jefferies, 2013; Lambon Ralph et al., 2016) or only the frontal regions (Binder and Desai, 2011). The functional assignments of specific roles to these regions are controversial and vary across models (Patterson et al., 2007; Binder and Desai, 2011; Jefferies, 2013; Lambon Ralph et al., 2016; Martin, 2016).

One important type of empirical evidence that was missing from the construction of a full neural model of semantic processing is the overall wiring structure, i.e., how the widely distributed semantic-related brain regions are topologically connected to support this complex faculty. Empirical profiling of the wiring pattern of the semantic system would not only provide direct evidence for how such diverse and distributed brain regions are communicated and incorporated but also pose important constraint on the understanding of the functions of individual regions, given that the functionality of a brain region is tightly related to its functional/structural connectivity patterns (Passingham et al., 2002). The importance of connectivity patterns has also been highlighted by previous models (Lambon Ralph et al., 2016; Martin, 2016), but only vague predictions about the global connectivity pattern could be derived: the modality/attribute-specific representations for a given concept are directly linked (Martin, 2016) or merged into higher-order representations in a graded manner (Lambon Ralph et al., 2016). The empirical evidence of the global wiring structure was absent, however, until recently.

The development of brain network analyses, advanced by the growing availability of techniques to measure brain connectivity and graph-theoretic approaches, offers a novel and global perspective to depict the topological organization of brain networks (Bullmore and Sporns, 2009; He and Evans, 2010; Sporns, 2013). A set of recent studies began to investigate the manner in which the widely distributed semantically relevant brain regions are connected, providing compelling clues about the organizational structure of the semantic system from a network viewpoint. This review seeks to highlight the recent empirical evidence about the network structure of the semantic system and to consider previous region-based studies in this new framework, leading to the proposal of a tri-network neurocognitive model of semantic processing. We will finally discuss how this network-based model generates new types of research avenues to study the neural basis of semantic processing.

## Semantic functional network: modules and hubs

### Brain networks and graph theory

The global topological structure of a complex system can be quantified using various graph-theoretic measurements. Under this framework, the brain can be modeled as a network incorporating nodes and edges. The nodes correspond to brain regions that can be defined as regions of interest, e.g., building spheres around peaks obtained through activation studies (Power et al., 2011, 2013; Vandenberghe et al., 2013; Xu et al., 2016) or according to anatomical landmarks (Salvador et al., 2005; He et al., 2009; Fang et al., 2015). The edges correspond to interregional connections that can be measured by multiple non-invasive imaging techniques, such as, diffusion tensor imaging tracking white matter tracts (Basser et al., 1994), or the resting-state functional connectivity reflecting intrinsic functional coupling (Biswal et al., 1995).

**Figure 1:**
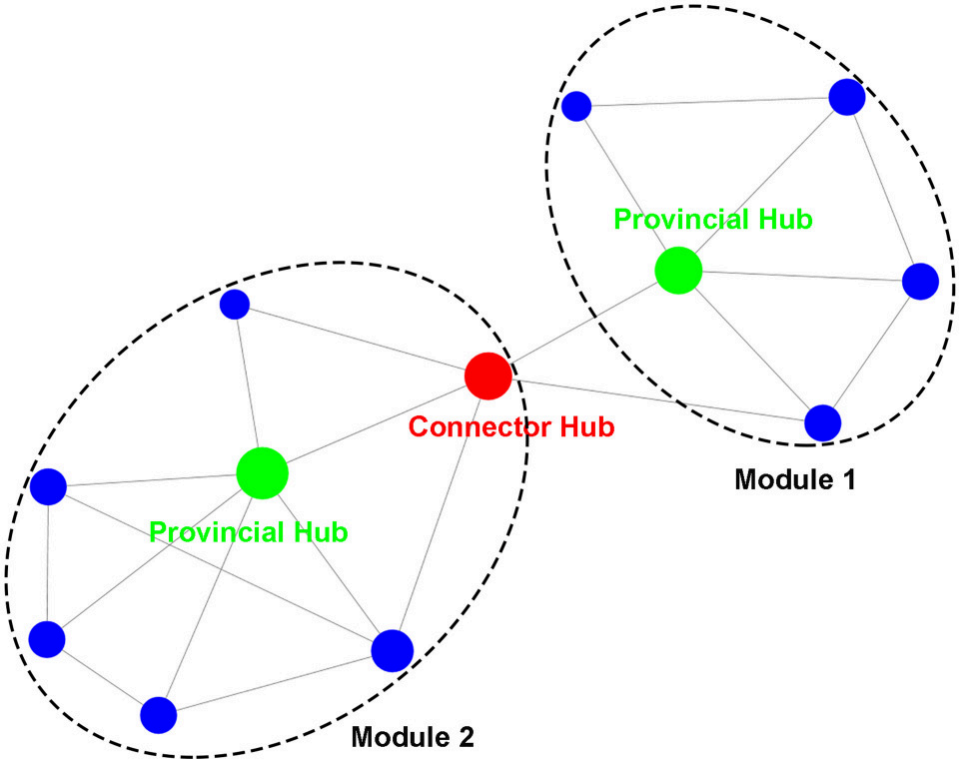
Illustrations of modules and hubs in a network. The modules with dense intra-community connections are identified within the dashed circles. The hubs are the nodes with high degrees (the number of connections that are maintained by a node) that are reflected by the size of nodes. Provincial hubs primarily connect nodes of their own module, while connector hubs are important in bridging different modules.

By applying graph theory, the topological properties of the brain network can be measured quantitatively (Bullmore and Sporns, 2009; He and Evans, 2010). Two important network structures are modules and hubs (Figure 1). Modules refer to a community of nodes with internal connections that are much denser than those between communities. Various algorithms can be used to detect a modular structure in a network (Fortunato, 2010; Sporns and Betzel, 2016), e.g., hierarchical clustering or information-based theory. Hubs refer to nodes that have central roles in network communication, commonly identified as nodes with densest connections (van den Heuvel and Sporns, 2013). The role of hubs can be also described in terms of their connectivity arrangements in a modular structure (Guimera and Amaral, 2005; He et al., 2009; Power et al., 2013); provincial hubs have connections primarily to the nodes of their own module, while connector hubs have relatively even connections to the nodes of the modules they connect.

Modular and hub structure can provide important clues about the functional segregation and integration among brain regions (Sporns, 2013). Modularity analyses have revealed that the whole-brain intrinsic functional network can be consistently partitioned into segregated modules that correspond to dissociable cognitive components in the human mind, e.g., visual, somatomotor, default mode, dorsal/ventral attention, and control (He et al., 2009; Power et al., 2011; Yeo et al., 2011). Connector hubs linking different modules have been found to be essential to the integration of multiple cognitive functions, as damage to these regions was found to cause severe and widespread cognitive deficits (Warren et al., 2014).

### Brain networks and the semantic system

**Figure 2:**
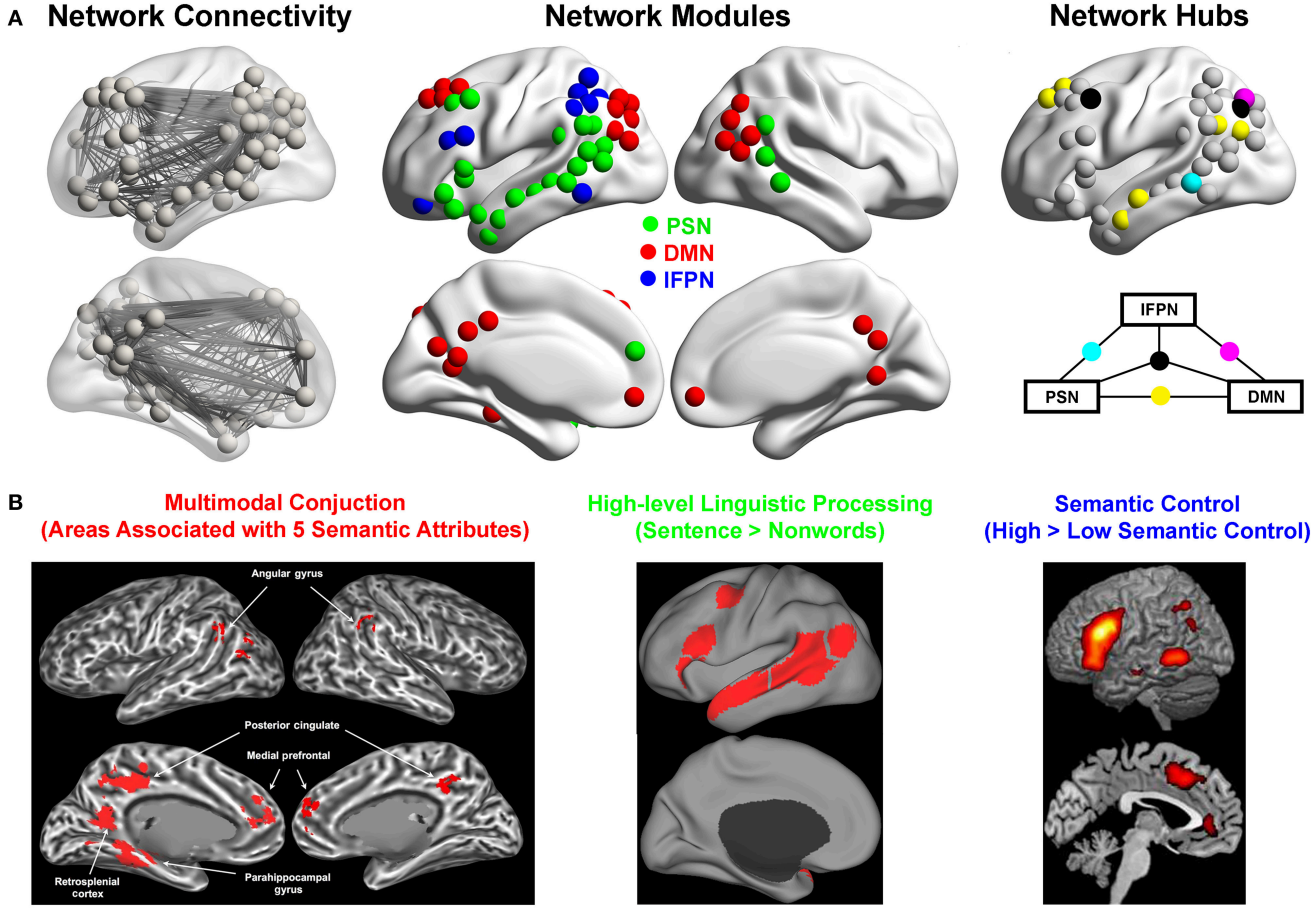
Semantic functional network: modules, hubs, and their cognitive functions. **(A)** The organization of the intrinsic functional network of semantic processing. Left: the semantic network showing nodes and edges, with nodes defined as the regions consistently activated during semantic processing obtained from a meta-analysis (Binder et al., 2009), and edges defined as the resting-state functional connectivity strength; Middle: the modules of the semantic network obtained by applying a graph-theoretic approach to the underlying connection patterns. Right: The connector hubs linking the three modules. Reproduced with permission from Xu et al. (2016). **(B)** Example results from task-evoked fMRI studies that shed light on the functions of the three modules. Left: The conjunction areas of five semantic aspects including shape, sound, motion, color, and manipulation from 900 words, which resemble the areas of Module DMN. Reprinted with permission from Fernandino et al. (2016); Middle: High-level linguistic processing regions generated from the group-level language localizer from 220 participants, which resemble the brain areas of Module PSN (https://evlab.mit.edu/funcloc/download-parcels); Right: The semantic control areas generated from a meta-analysis of 53 studies, which resemble the areas of Module lFPN. Reproduced with permission from Noonan et al. (2013).

The global topological structure of the semantic system was recently investigated. One study (Xu et al., 2016) constructed the intrinsic functional semantic network, with nodes defined as regions obtained from a careful and comprehensive meta-analysis (Binder et al., 2009) and edges defined as the inter-regional resting-state functional connectivity (Figure 2A, left). The meta-analysis results that were used to define nodes were based on 120 task-evoked neuroimaging studies that contain 187 semantic contrasts with orthographic and phonological processing demands and task difficulty matched for. The graph-theoretic approach was applied on this network, revealing three segregated modules (Figure 2A, middle), which were highly stable across datasets and various network construction methods (e.g., different nodal resolution—voxels or regions). According to the anatomical layout, these three modules were labeled as the left perisylvian network (PSN), the default mode network (DMN), and the left frontoparietal network (lFPN). Connector hubs that integrate different modules were also identified, e.g., the ATL was found to be the connector hub linking Modules DMN and PSN, while the posterior middle temporal gyrus (pMTG) was identified as the connector hub linking Modules DMN and FPN (Figure 2A, right). A similar approach was employed in a study (Fang et al., 2015) that constructed the structural semantic network by correlating the integrity of white matter tracts with the semantic performances in patients with brain damage. Although it was acknowledged that this constructed semantic structural network may not be complete owing to restricted lesion distributions (lack of posterior lesions), three modules were obtained in this study that aligned with those found in the intrinsic functional network (Xu et al., 2016): the “medial temporal lobe module” in the structural network functionally coincided with the Module DMN; the “orbital frontal–temporal/occipital module” overlapped with the Module PSN; the “opercular/triangular/middle frontal–subcortical module” corresponded to the Module FPN. Note that there was only one study that investigated the semantic-task effects on the connectivity patterns (Vandenberghe et al., 2013). They first identified regions that were activated during an associative semantic task (the Pyramids and Palm Trees test). They then examined the functional connectivity pattern among these regions during the semantic and visuoperceptual control conditions. Six modules were detected, including one anatomically corresponding to the classical perisylvian language system, one to the visual perception system, and the other four that were difficult to label. Given that the functional connectivity was established based on both semantic and perceptual blocks, it was difficult to conclude whether the network structures were related to semantic or perceptual processing or both. Indeed, the visual module might be due to the visual tasks being employed and the observed perisylvian module converged onto the PSN module within the semantic system identified during the resting state (Xu et al., 2016).

Although there are only these few studies that directly addressed the global topological structure of the semantic system, clues about the semantic network structure could be gleaned from several other lines of researches. First, several recent studies focused on the connectivity pattern of specific semantically-related regions as seeds, such as, the posterior and the middle part of the MTG (Turken and Dronkers, 2011; Wei et al., 2012; Davey et al., 2015, 2016; Feng et al., 2015), the ATL (Turken and Dronkers, 2011; Binney et al., 2012; Pascual et al., 2013; Feng et al., 2015; Jackson et al., 2016), the angular gyrus (Davey et al., 2015), the orbital and triangular part of the inferior frontal gyrus (IFG) (Saur et al., 2008; Turken and Dronkers, 2011; Feng et al., 2015), and the fusiform gyrus (Saur et al., 2008). They found that these regions have rich functional or structural connections with each other, and provide fragmented yet illuminating views about the overall patterns of the whole system, which converge with the network-level findings above. For example, consistent with the findings that the pMTG was the connector-hub between Modules PSN and FPN, seed-based studies showed that the pMTG has functional/structural connections with the brain areas in Modules PSN and Module FPN like the lateral temporal cortex, the IFG, the intraparietal sulcus (Turken and Dronkers, 2011; Wei et al., 2012; Davey et al., 2015, 2016; Feng et al., 2015). Also the seed-based studies found that the lateral ATL was functionally/structurally connected with the brain areas within the Modules DMN and PSN, such as, the ventral and anterior part of the IFG, the AG and the precuneus (Binney et al., 2012; Pascual et al., 2013; Feng et al., 2015; Jackson et al., 2016), consistent with the topological findings that the lateral ATL was the connector-hub between Modules PSN and DMN. Second, patient studies have focused on specific white-matter connections and revealed that disruptions in several large white-matter tracts, including the inferior fronto-occipital fasciculus, the anterior thalamic radiation, and the uncinate fasciculus that connect left temporal, frontal, parietal, and subcortical regions, are associated with semantic deficits (Duffau et al., 2005; Agosta et al., 2010; Acosta-Cabronero et al., 2011; Han et al., 2013). These results are in line with the rich intrinsic functional connections illustrated in Figure 2A (left). Finally, results about the whole-brain global network structure also tend to be in accord with the results focused only on semantic regions (He et al., 2009; Power et al., 2011; Yeo et al., 2011).

Intriguingly, the tri-module network structure is not naturally predicted or accounted for by any of the existing models of semantic processing, as outlined above. This network structure suggests the need to consider the functions of the semantic-related regions in the framework of the three modules—whether regions belonging to the same module have homogenous functions and what those functions might be. Notably, the existence of brain modular structure does not directly imply cognitive dissociation or synthesis—this is the classical reverse inference fallacy. Nonetheless, the cognitive hypotheses of semantic processing provide natural clues for interpreting the function of the brain network structure and together help formulate comprehensive neurocognitive models (Henson, 2005; Price and Friston, 2005; Poldrack, 2006). In the following section, we will review task-evoked neuroimaging and neuropsychological evidence about the functions of the brain regions in these three modules (summarized in Table 1) and postulate the functions of each module accordingly based on a broad range of pathological and functional neuroimaging data and existing meta-analyses results.

**Table 1:** Regional task-based neuroimaging and stimulation results of brain areas in the semantic system, organized by the three network modules and connector hubs revealed by the graph-based analyses.

**Semantic components**	**Empirical findings**			**Semantic modules**			**Semantic hubs**			
	**Properties**	**Methods^a^**	**Tasks/Contrasts^b^**	**DMN**	**PSN**	**lFPN**	**ATL**	**pMTG**	**pIPS**	**AG^c^**
Multimodal experiential	Memory-based simulation	Activation	Navigation, Prospection, Autobiographical Memory Retrieval, Theory of Mind (**Meta-analysis**^e^**:** Spreng et al., 2009)	Spreng et al., 2009			Spreng et al., 2009			Posterior Parts: Spreng et al., 2009
	Multimodal integration	Connection	Converging Areas Tracing Functional Connectivity From Multiple Modality-specific Areas (Sepulcre et al., 2012)	Sepulcre et al., 2012			Sepulcre et al., 2012			Sepulcre et al., 2012
		Activation	Activation Overlaps for Attributes of Color, Motion, Shape, Sound, and Manipulation (Fernandino et al., 2016)	Fernandino et al., 2016						Fernandino et al., 2016
	Modulation by the richness of experience	Activation	Concrete − Abstract (Binder et al., 2005; Sabsevitz et al., 2005; Wang et al., 2010; Hoffman et al., 2015) (**Meta-analysis:** Wang et al., 2010) Famous − General (Sugiura et al., 2006; Wang et al., 2016) Personal − Unfamiliar (Sugiura et al., 2006)	Binder et al., 2005; Sabsevitz et al., 2005; Sugiura et al., 2006; Wang et al., 2010, 2016; Hoffman et al., 2015			Medial, Ventral and Lateral Parts: Sugiura et al., 2006; Hoffman et al., 2015; Wang et al., 2016			Posterior Parts: Binder et al., 2005; Sabsevitz et al., 2005; Wang et al., 2010; Hoffman et al., 2015
Language supported	Linguistic specificity	Activation	Sentences − Nonword lists Not in Contrasts of Arithmetic, Working memory, Control, and Music (Fedorenko et al., 2011)		Fedorenko et al., 2011		Fedorenko et al., 2011	Fedorenko et al., 2011		Fedorenko et al., 2011
	Verbal and nonverbal semantic processing	TMS	Word Semantic Association − Perceptual Association (Pobric et al., 2010; Hoffman et al., 2012) Picture Semantic Association − Perceptual Association (Pobric et al., 2010; Hoffman et al., 2012) Synonym Judgment − Number Judgement (Pobric et al., 2007) Category Decision − Phonologic Decision (Hartwigsen et al., 2015)		Pobric et al., 2007, 2010; Hoffman et al., 2012; Hartwigsen et al., 2015		Pobric et al., 2007, 2010	Hoffman et al., 2012		Hartwigsen et al., 2015
	Modulation by the dependency of linguistic associations	Activation	Abstract − Concrete (Binder et al., 2005; Sabsevitz et al., 2005; Wang et al., 2010; Hoffman et al., 2015) (**Meta-analysis**: Wang et al., 2010) Idiomatic − Literal (Lauro et al., 2008; Boulenger et al., 2009)		Binder et al., 2005; Sabsevitz et al., 2005; Lauro et al., 2008; Boulenger et al., 2009; Wang et al., 2010; Hoffman et al., 2015		Lateral and Dorsal Parts: Binder et al., 2005; Sabsevitz et al., 2005; Lauro et al., 2008; Boulenger et al., 2009; Wang et al., 2010; Hoffman et al., 2015	Sabsevitz et al., 2005; Lauro et al., 2008; Boulenger et al., 2009; Hoffman et al., 2015		Anterior Parts: Lauro et al., 2008; Hoffman et al., 2015
Semantic control	Modulation by the difficulty of semantic tasks^d^	Activation	High − Low Semantic Control (**Meta-analysis:** Noonan et al., 2013)			Noonan et al., 2013		Noonan et al., 2013		Dorsal Parts: Noonan et al., 2013
		TMS	Weak − Strong Semantic Association (Whitney et al., 2011, 2012) Attribute − Global Semantic Association (Whitney et al., 2012) Cyclical Picture Naming of Semantic-related Sets − Semantic-unrelated Sets (Krieger-Redwood and Jefferies, 2014) Identity Matching of Word-picture Associations at Superordinate Level − Specific Level (Davey et al., 2015) Thematic Matching of Week − Strong Word Picture Associations (Davey et al., 2015)			Whitney et al., 2011, 2012; Krieger-Redwood and Jefferies, 2014		Whitney et al., 2011, 2012; Krieger-Redwood and Jefferies, 2014; Davey et al., 2015	Whitney et al., 2012	Dorsal Parts: Whitney et al., 2012
		Activation	Attribute − Global Semantic Association (Badre et al., 2005; Davey et al., 2016)			Badre et al., 2005; Davey et al., 2016			Badre et al., 2005; Davey et al., 2016	Dorsal Parts: Badre et al., 2005; Davey et al., 2016

a *Activation is the method using univariate analysis in fMRI or PET studies; connection is the method using functional connectivity in fMRI studies*.

b *For the method of activation, we list the tasks or contrasts that induce activation of specific modules or hubs; for the method of TMS, we list the tasks or contrasts that were disrupted while TMS to regions in specific modules or hubs*.

c *Also containing areas around the AG*.

d *For the contrasts, the former tasks are assumed to require greater control demand than the latter*.

e *The meta-analyses studies are highlighted in bold*.

## Segregated brain modules, segregated semantic components

### The DMN: the multimodal experiential system

This module (red nodes in Figure 2A, middle) encompasses bilateral retrosplenial gyri/precuneus, bilateral medial prefrontal cortices, bilateral posterior angular gyrus (AG) extending to the superior division of the lateral occipital cortex, the left superior frontal gyrus (SFG), and the middle part of the left fusiform cortex/parahippocampal gyrus. These are the core regions of the DMN, originally defined as a brain system showing task-induced deactivation (Raichle et al., 2001).

The striking resemblance between the DMN and the semantic processing regions has long been noticed (Binder et al., 1999, 2009; Binder, 2012; Wei et al., 2012). Compared to the resting state, the DMN is significantly less deactivated for semantic tasks compared to perceptual or phonological tasks (Binder et al., 1999, 2009; Seghier et al., 2010; Wirth et al., 2011; Humphreys et al., 2015). Why is semantic processing special? One view is that the DMN does not process semantics and its activation during semantic processing is only the epiphenomenon of lower attention demand of semantic tasks relative to other tasks (Humphreys et al., 2015). In line with this view, the DMN plays a general intrinsic role, serving to maintain a functional balance with brain systems engaged in attention and control (Raichle, 2015). The magnitude of the DMN deactivation in visual tasks is related to the degree of task demand (Singh and Fawcett, 2008), and functional spontaneous fluctuations of the DMN was anti-correlated with the top-down attention network (Fox et al., 2005; Chai et al., 2012). Given that the DMN activation in semantic tasks was observed even when the task demand (defined by reaction time) of semantic processing is matched to (Wirth et al., 2011) or even stronger than (Seghier et al., 2010) the control tasks or when the task demand was explicitly regressed out (Binder et al., 2005), we suspect that the engagement of the DMN in semantic processing is not only due to the effects of general difficulty.

Another view is that DMN functionality is related to semantics. It has been considered that the resting state is not a blank state but rather involves “spontaneous cognitions,” such as, remembering the past and thinking about the future, in which the DMN is recruited (Andrews-Hanna et al., 2010). Meta-analyses reveal that the DMN is the shared neural foundation of a spectrum of cognitive tasks, e.g., autobiographical memory retrieval, prospection, theory of mind, and navigation (Spreng et al., 2009). Considering the common cognitive component of these tasks, the DMN was considered as a memory-based simulation system, serving to piece together materials from one's past experience to construct new scenes or context, which can be self-projected into for evaluation, prospection, and mentalizing (Buckner and Carroll, 2007; Hassabis and Maguire, 2007; Schacter et al., 2007; Buckner et al., 2008). It has been discussed that semantic processing is a necessary component underlying such processes (Binder et al., 1999, 2009; Binder and Desai, 2011; Binder, 2012).

We wish to bring attention to another intriguing aspect of the DMN: many of its constituent regions are where information from multiple modalities converges. Using a “step-wise functional connectivity” approach to trace information pathways from unimodal regions to higher-order convergence zones, areas in the DMN were found to be the final stable state where information pathways from all modalities reach (Sepulcre et al., 2012). Applying a “parametric modulation” method to decompose the activation of a word into effects of multiple modality-specific attributes, areas where all the attribute effects overlapped largely fall in the DMN (Fernandino et al., 2016) (Figure 2B, right). This evidence suggest that this system is likely to support the integration of simulation-based multimodal experiential representation. Using the earlier “Beijing” example, we can use our experience to construct multimodal scenarios about what “Beijing” entails, e.g., the views of the Forbidden City, the taste or smell of a Beijing roast duck, or the rhotic vowels of the Beijing dialect. As concepts acquired from rich personal experience can be more automatically instantiated through this approach, the DMN is more strongly activated by concrete (Binder et al., 2005; Sabsevitz et al., 2005; Wang et al., 2010; Hoffman et al., 2015), famous (Sugiura et al., 2006; Wang et al., 2016), and personal (Sugiura et al., 2006; Renoult et al., 2012) concepts, in contrast with abstract, common, and general concepts, respectively. Patients with Alzheimer's disease or mild cognitive impairment in which the DMN is usually compromised tended to recall past events divested of rich sensory-perceptual imagery (Irish et al., 2011), and show deficits of knowledge of famous people and their physical features (Borg et al., 2010).

However, the DMN is neither sufficient nor necessary for all semantic tasks. Unlike damage to the PSN (see below), damage to brain areas of the DMN in patients with Alzheimer's disease (Nestor et al., 2006) or in patients suffering a stroke with lesions encompassing the posterior cingulate cortices (Leech and Sharp, 2014) or the parietooccipital cortex (Berryhill et al., 2007) seems not to cause severe deficits in semantic tasks that mostly probe association or function knowledge. We thus contend that the DMN hosts the aspects of semantic knowledge that are tightly related to multimodal experiences and is not necessary for those tasks that do not require the retrieval of specific attributes based on such experiences (e.g., associating “Beijing” with “China” does not require retrieval of the specific location or landscape of Beijing, and “fox” with “shrewdness” does not require retrieval of what a fox's ears look like).

### The PSN: the language-supported semantic system

This module (green nodes in Figure 2A, middle) includes the entire length of the left middle temporal gyrus, the ventral part of the left IFG, and the junction area of the left posterior temporal and inferior parietal lobes (the left temporoparietal junction). A common characteristic of these regions is that they together fit well with the language network (Figure 2B, middle) (Friederici, 2011), which shows selective activation by sentences in contrast to nonword lists and not by multiple non-linguistic tasks, such as, arithmetic, working memory, cognitive control, or music (Fedorenko et al., 2011). For semantic processing, the left ventral IFG and the left temporal cortex in this module were consistently found to be more strongly activated by abstract and idiomatic concepts with meanings that presumably rely heavily on linguistic associations (Hoffman, 2015) than by concrete (Binder et al., 2005; Sabsevitz et al., 2005; Wang et al., 2010; Hoffman et al., 2015) or literal (Lauro et al., 2008; Boulenger et al., 2009) terms. Intriguingly, lesions or atrophies in regions of this system affect semantic comprehension in not only verbal but also nonverbal tasks using picture, sound or motion as inputs, e.g., the anterior temporal cortex (Bozeat et al., 2000; Mummery et al., 2000; Garrard and Carroll, 2006; Jefferies and Lambon Ralph, 2006; Robson et al., 2012), the left posterior temporal and temporoparietal cortices (Jefferies and Lambon Ralph, 2006; Corbett et al., 2009; Robson et al., 2012; Thompson et al., 2015). Transcranial magnetic stimulation (TMS) to the left (ATL) (Pobric et al., 2007, 2010) and the left posterior middle temporal gyrus (pMTG) (Hoffman et al., 2012) impedes semantic performances in both verbal and nonverbal tasks without affecting non-semantic tasks of comparable difficulty.

What kind of function would be relevant for linguistic processing and for semantic processing in both verbal and nonverbal tasks? We postulate that this module supports (amodal) semantic representation that is embedded in the language system. Given the paucity of research on language-related dimensions in the neural semantic space, the exact nature, content, or format of representation that is supported by the PSN remains unknown. The point here is that the identification of a PSN module (segregated from the DMN) in the semantic brain network suggests a natural candidate system for a kind of representation distinct from experiential-based representations. There has been much discussion in the cognitive, psycholinguistic and artificial intelligence fields about how linguistic contexts (e.g., word association, word order, and syntactic structure) contribute to representing meaning (Landauer and Dumais, 1997; Burgess, 1998; Jones and Mewhort, 2007; Barsalou et al., 2008; Dove, 2009, 2010; Vigliocco et al., 2009; Mikolov et al., 2013a, 2014), and relevant hypotheses should be articulated for testing against neural responses in the PSN. One simple possibility is that the occurrence patterns in natural language differ from the objects and events associations in the real-word scenes at least to some extent, and such language-occurrences modulate the experience-based relations among concepts and create new types of relations. That is, the specific association patterns due to the linguistic contexts, among lexical representations and/or semantic representation in the DMN system, may give rise to information that is part of the semantic representation. There are two important points to note. First, while the symbolic accounts of semantic representation may satisfy this description, the representational format in the Module PSN is not necessarily amodal symbolic. Second, there is a long debate about the necessity of having “lexicalized concepts” being different from “prelinguistic concepts” (Caramazza, 1997; Levelt et al., 1999; Vigliocco and Vinson, 2007). In the spirit of parsimony, we do not think having a separate lexicalized concepts here are necessary. The semantic information supported by the language system could be coded in the association patterns of lexical representations themselves, which points to “prelinguistic” concepts (the experiential representations in the Module DMN). Importantly, such language-supported knowledge constitutes an integral aspect of semantics (consider the knowledge given by “Beijing is the capital of China” for “Beijing”), and disrupting this module would lead to impairment for semantic tasks requiring this type of knowledge in not only verbal but also nonverbal semantic tasks.

### The lFPN: the semantic control system

This module (blue nodes in Figure 2A, middle), including the dorsal part of the left IFG, the IPS, and a region in the posterior inferior temporal lobe. It is largely similar to the left hemisphere part of a broader bilateral frontoparietal control system revealed by whole-brain intrinsic functional connectivity analyses (Vincent et al., 2008; Power et al., 2011; Yeo et al., 2011). The broader frontoparietal control network acts as a flexible hub (Cole et al., 2013), offering rapid adaptive coordination of other functional systems in a task- and time-appropriate fashion (Dosenbach et al., 2008). While the right part of this network is involved in sensorimotor-related control (Levy and Wagner, 2011; Harel et al., 2014), the left one is more engaged in the conceptual and linguistic domains (Noonan et al., 2013; Harel et al., 2014). Compared to the FPN that has been referred to a multi-demand system that is activated during a wide variety of demanding cognitive tasks (Duncan, 2010; Fedorenko et al., 2013), the FPN that is most consistently associated with semantic control is more left-lateralized with its frontal part being more posterior and inferior.

The proposal that regions in this module serve a control role in semantic cognition, i.e., semantic control, has been discussed in depth in recent reviews (Jefferies, 2013; Lambon Ralph et al., 2016). We will not reiterate all the relevant empirical evidence here but will refer to a few lines of representative evidence (Table 1). Meta-analyses show that brain areas in the lFPN are more strongly activated by semantic tasks requiring greater semantic control (Noonan et al., 2013) (Figure 2B, right). Attribute semantic tasks, which require attention on memory images of specific attributes (e.g., color, shape, manipulation), induce stronger activation in this module, in contrast to semantic tasks, which do not (Badre et al., 2005; Davey et al., 2016). Lesions extending to the lFPN lead to so-called “semantic access deficits” (Mirman and Britt, 2014), with sensitivity to the semantic distance and the strength of competitors in semantic association tasks (Noonan et al., 2010), refractory effects (Jefferies et al., 2007; Thompson et al., 2015), and item or task inconsistency across different semantic tasks (Jefferies and Lambon Ralph, 2006; Corbett et al., 2009; Robson et al., 2012). TMS to the triangular part of the left IFG selectively disrupts semantic tasks with higher executive demands without affecting those with low demand or non-semantic tasks and to the left IPS disrupts performance in attribute semantic tasks (Whitney et al., 2011, 2012; Krieger-Redwood and Jefferies, 2014). In the context of the three-module structure, this system “controls” the semantic content represented in the other two modules—retrieving the specific experiential attributes in the DMN and the language-based knowledge in the PSN—according to current task demands. Regions in this system are more strongly activated by semantic tasks than by other tasks that presumably also need control (Binder et al., 2009), either because semantic processing requires stronger/more complex controls or because these regions are more strongly connected with other semantic representation components and are more visible in semantic tasks.

## Integrated hubs, integrated semantic components

The three modules must be integrated for a given semantic task. When we hear the word “Beijing” or see a picture of Beijing in various linguistic or real-world contexts, multiple aspects of semantic knowledge and the control systems are activated to achieve understanding. How are these three modules integrated? The network analysis identified a series of connector hub regions that are important in linking the three modules discussed above (Figure 2A, right) (Xu et al., 2016): the left ATL linking Modules PSN and DMN, the left pMTG linking Modules PSN and lFPN, the left posterior intraparietal sulcus (pIPS) linking Modules DMN and lFPN, and the left AG and the border areas of the superior and middle frontal gyri (left SFG/MFG) linking all three brain systems. Note that these regions were also discussed above in the three modules; all regions are assigned to a module, even regions with relatively evenly distributed connections with multiple modules (i.e., connector hubs). These regions, especially the ATL (Patterson et al., 2007; Lambon Ralph et al., 2016), the pMTG (Wei et al., 2012; Davey et al., 2016), and the AG (Schwartz et al., 2011; Bonner et al., 2013; Seghier, 2013; Price et al., 2015), have been considered to be the “hub” regions of the semantic system, motivated by various types of evidence about their importance in semantic processing. Based on the literature, multiple types of semantic functions, including the hypothesized ones of the corresponding networks they link, have been reported in these regions (Table 1).

The connectivity-based findings reviewed here provide direct empirical evidence for their (connector-) “hub” status, the definition of which is based on the connectivity patterns (Guimera and Amaral, 2005; van den Heuvel and Sporns, 2013), and revealed that they differ in terms of the systems they connect. For instance, the ATL is where multimodal experiential representation and language-supported representation meet, whereas the pIPS and the pMTG are where the control system interacts with the experiential and language-supported representations, respectively. These findings that derived from the topological patterns of these hub regions are in accordance with some previous notions about these regions that was inferred from regional activation patterns, e.g., that the ATL is the “transmodal” site between experiential and language-supported representations (Rogers et al., 2004; Patterson et al., 2007; Visser et al., 2010; Lambon Ralph, 2014; Rice et al., 2015), the IPS is associated with top-down attention to memory images (Cabeza et al., 2008), and the pMTG is the area for “controlled semantic retrieval” (Badre et al., 2005; Schwartz et al., 2011; Davey et al., 2015).

Notably, while the connectivity profiles suggest that they are likely to be the sites where different components of semantic processing are integrated, there are at least two possibilities about whether and how they actually merge. One is that they simply host adjacent yet different sub-regions belonging to different networks and with distinct functionalities. Another possibility is that they perform a similar function to different inputs within different tasks or some type of higher-order computations that merge the functions of multiple modules. In light of the network structure, studies on the nature of representation and processing supported by these regions should take into consideration the functions of the multiple networks they merge.

## A tri-network neurocognitive model of semantic processing

**Figure 3:**
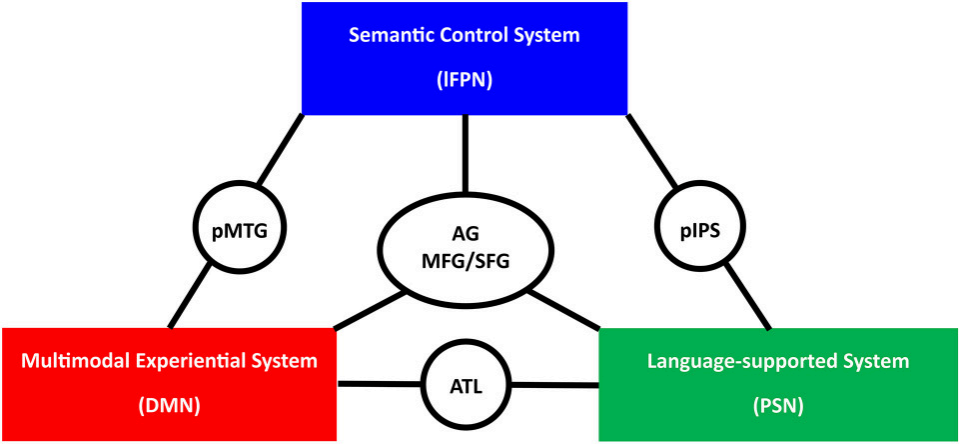
The schematic presentation of the tri-network neurocognitive model of semantic processing. lFPN, left frontoparietal network; DMN, default mode network; PSN, perisylvian network; pMTG, posterior middle temporal gyrus; ATL, anterior temporal lobe; pIPS, posterior intraparietal sulcus; AG, angular gyrus; SFG/MFG, superior and middle frontal gyrus.

Our review of recent brain network studies on semantic processing and consideration of previous region-based results in light of these new findings lead to the proposal of a tri-network neurocognitive model of human semantic processing (Figure 3): the widely distributed semantic regions are wired into three separate neural networks, which are likely to support three different cognitive components of semantic processing. The DMN serves as the multimodal experiential system, where experience-based knowledge across multiple modalities is integrated (e.g., the integration of various types of experiences one has with Beijing). The left PSN serves as the non-experiential system, where semantic content being supported by linguistic contexts are represented (e.g., “Beijing is the capital of China”). The lFPN serves as the semantic control system, acting on the other two modules for the retrieval of semantic knowledge in a task- and time-appropriate fashion. Semantic processing entails the coordination of these functional modules, which is likely to be achieved via a series of connector hubs in the ATL, the pMTG, the pIPS, the AG, and the SFG/MFG.

This tri-network model is mainly motivated by the modular and hub structure of the widely distributed semantic network and shares several key points with previous semantic neurocognitive models. The role of the ATL in binding various modalities (verbal and non-verbal) converges with the “hub and spoke” model as well as its recent update—the controlled semantic cognition model (Patterson et al., 2007; Lambon Ralph et al., 2016). The relevance of this distributed system in representing abstraction of modality-specific attributes/experiences is shared by the embodied-abstraction model (Binder and Desai, 2011). The distinction between representation and control is also well in line with these recent models (Binder and Desai, 2011; Jefferies, 2013; Lambon Ralph et al., 2016).

However, there are several important differences. Within representation, our current framework entails two segregated systems: multimodal experiential content in the DMN and language-supported content in the PSN. Both are “abstracted” away from modality-specific “embodiment,” but the degree of abstraction and the principle of abstraction are likely to differ between these two systems—one originated from real-world experience and one from language. In the DMN, the sensory, motor, and affective inputs from multiple modality-specific systems converge together to capture high-order conceptual representations (e.g., taxonomic categories or a whole event), which best fill the role of the “crossmodal conjunctive representation” proposed by Binder (2016). In the PSN, the meaning is supported by language contexts. The previous neuroanatomical semantic models have not articulated such potential differences between language-supported and experience-based semantic representations. In some models, only modality/property-specific representations were considered (Martin, 2016). In the hub-and-spoke/controlled semantic cognition model, language is just one modality of processing in parallel to other modalities (e.g., vision, sound, and valence) (Patterson et al., 2007; Lambon Ralph et al., 2016). In the embodied-abstraction model, abstraction happens from the modality-specific representations (Binder et al., 2009; Binder and Desai, 2011). Our framework incorporates evidence both from the network structure and regional studies using lesion and neuro-activation approaches, highlights the two distinct brain systems for two different types of semantic representation, which is more similar to the dual coding cognitive models of meaning (Paivio, 1986; Barsalou et al., 2008; Mahon and Caramazza, 2008; Dove, 2009, 2010; Vigliocco et al., 2009; Zwaan, 2014; Reilly et al., 2016), and provides specific dissociable target brain systems as the corresponding neural bases. A series of hub regions are also explicitly postulated to integrate the two kinds of semantic representation and to interact with the control system.

## Future perspectives

This tri-network model frames several lines of new questions about the brain basis of semantics. The central point is that from any of the various approaches, instead of studying the functionality and mechanisms of widely distributed cortical regions implicated in semantic processing individually, it would be more productive to study them in the contexts of the three sub-systems, examining both the modules as whole units and the roles of specific constituents (regions and connections). A few examples are outlined here. First, regarding different kinds of semantic processing, it remains to be understood how the connectivity pattern across different modules is configured according to different types of semantic tasks (e.g., with different semantic contents and difficulty levels). Would the configuration of connectivity patterns complement the findings of cortical representations? Specifically, would the connectivity within a particular module be specifically strengthened when the task involved more of the corresponding semantic contents (e.g., the Module PSN in a task that requires to process abstract concepts)? Would the connectivity between the Module FPN and the other two modules be strengthened when the task was more difficult? Second, about the different types of semantic content and encoding mechanisms (experiential vs. those supported by linguistic contexts), the DMN and the PSN modules in the semantic system provide the candidate target brain systems to test their distinctions and interactions. Cognitive models built from experience-based attributes and those from various natural language processing models, e.g., the Latent Semantic Analysis (Landauer and Dumais, 1997) or the neural network models such as, word2vec (Mikolov et al., 2013a, 2014), could be compared against neural activity patterns in these two neural modules. Will activity pattern and/or connectivity pattern across the DMN and the PSN modules correlate relatively more strongly with the semantic space generated according to experiential and the linguistic contextual models, respectively? Third, from a developmental perspective, it would be intriguing to see during semantic knowledge acquisition whether the neural representational patterns of different sub-systems are modulated by corresponding types of experience (linguistic vs. experimental). Finally, new questions emerge about the nature of computation at the connector-hub regions, i.e., how information across multiple modules is integrated. Do the hub regions simply host sub-regions with distinct functionalities (exhibiting distinct neural representational pattern) or do they perform some type of higher-order computation that merges the functions of multiple modules (exhibiting high-order neural representational patterns)?

## Author contributions

All authors listed have made a substantial, direct and intellectual contribution to the work, and approved it for publication.

### Conflict of interest statement

The authors declare that the research was conducted in the absence of any commercial or financial relationships that could be construed as a potential conflict of interest.
